# Chylothorax in a patient with advanced ovarian cancer: a case report and literature review

**DOI:** 10.3389/fonc.2025.1555038

**Published:** 2025-09-30

**Authors:** Qiuying Wang, Xiaolan Zhang, Ke Niu, Wenjie Shen

**Affiliations:** Senior Department of Obstetrics & Gynecology, the Fourth Medical Center of PLA General Hospital, Beijing, China

**Keywords:** chylothorax, chylous ascites, ovarian cancer, diagnosis, treatment

## Abstract

**Background:**

Ovarian cancer complicated with chylothorax is extremely rare, and only eight papers can be searched in PubMed. We report a case of advanced ovarian cancer with extensive lymphatic metastasis that led to chylothorax and then chylous ascites. Combined with the literature review, we try to identify its clinical characteristics.

**Case description:**

A 64-year-old patient with advanced ovarian cancer relapsed, presenting with extensive lymphatic metastasis and a right pleural effusion. Thoracentesis was performed. The drainage fluid had a milky white appearance with triglyceride levels >1.24 mmol/L (110 mg/dL), a cholesterol level <5.18 mmol/L (200 mg/dL), and a positive chyle test. A multi-disciplinary team recommended anti-tumor therapy for ovarian cancer and conservative treatment of chylothorax, including a strict diet with low-fat medium–long-chain triglycerides and chest tube insertion with interleukin-2 intrapleural perfusion as the pleurodesis agent. Chylothorax improved significantly with progressively reduced and transparent pleural effusion. However, strict diet control affected her quality of life negatively, and the patient was not able to adhere to the diet plan. Therefore, chylothorax recurred, but chyle was produced slowly; thoracentesis was performed repeatedly with IL-2 or cisplatin pleural perfusion at intervals of 2–5 months (a total of six times) in March 2021–June 2023. In June 2023, the patient was not able to tolerate systemic chemotherapy because of severe myelosuppression after fourth-line chemotherapy. The patient developed bilateral chylothorax and then chylous ascites in December 2023. Finally, the patient died in March 2024.

**Conclusion:**

Ovarian cancer complicated with chylothorax is a rare condition with a relatively poor prognosis. Pleural fluid analysis can identify this condition when clinical suspicion exists. Anti-tumor treatment of ovarian cancer and conservative management of chylothorax were first recommended instead of surgery, which have been proven to be effective in treating OCC.

## 1 Introduction

Pleural effusion is common in advanced ovarian cancer, but ovarian cancer with chylothorax (OCC) is extremely rare; only seven papers can be searched in PubMed (1–7). Ovarian cancer with chylothorax and chylous ascites was reported only in one paper. Different from pleural effusion, OCC has its own particularity in clinical features and treatment. We report a case of advanced ovarian cancer with extensive lymphatic metastasis that led to chylothorax and then chylous ascites. Combined with the literature review, we try to identify the characteristics of the disease.

## 2 Case description

A 64-year-old woman presenting with abdominal distension and loss of appetite for 1 month was referred to our hospital in May 2018 (**Table 1**). Physical examination revealed massive abdominal distension with free fluid in the abdominal cavity. Contrast-enhanced computed tomography (CT) showed multiple cystic solid mass lesions with rich blood supply in the pelvis, the largest approximately 18 × 15 cm; multiple enlarged retroperitoneal lymph nodes (up to 5 cm in diameter); enlarged mediastinal lymph nodes; massive ascites; and bilateral pleural effusion (9.2 and 3.9 cm in depth in the left and right, respectively). The serum carbohydrate antigen 125 (CA-125) was 1,539 U/mL. Abdominal puncture and thoracentesis were performed. Both ascites and pleural fluid were serous and yellow in appearance. Adenocarcinoma cells were found on cytology from both ascites and pleural fluid. Ovarian epithelial cancer was diagnosed in stage IV. Neoadjuvant chemotherapy with paclitaxel and carboplatin was given for three cycles with interleukin-2 left-sided chest perfusion once (from May 2018 to October 2018). Both ascites and pleural fluid disappeared after one course of chemotherapy. An optimal intermediate cytoreductive procedure was performed with residual lesions <1 cm (R1) on November 13, 2018. Histopathology result showed moderately differentiated (G2) serous papillary adenocarcinoma in both ovaries, diffuse metastatic adenocarcinoma nodules in the omentum, and multiple metastases existing in the para-aortic lymph nodes and bilateral pelvic lymph nodes (4/6, 13/22, and 14/15, respectively). Four courses of chemotherapy were given after the operation (from December 2018 to February 2019).

**Table 1:** The timeline from the initial diagnosis to death.

Event	Date
Initial diagnosis	May 2018
Initial treatment	May 2018 to February 2019
The first recurrence (lymph node enlargement)	May 2020
Recurrence treatment	August 2020 to December 2020
Chylothorax (the second recurrence)	March 2021
The second recurrence treatment	March 2021 to October 2021
Maintenance treatment	November 2021 to May 2022
Third-line/fourth-line treatment (increased CA-125, lymph node enlargement, and chylothorax)	June 2022 to June 2023
Chylous ascites	December 2023
Death	March 2024

CA-125, carbohydrate antigen 125.

In May 2020 (15 months after the last chemotherapy), an enlarged lymph node (8 cm in diameter) was palpable in the left neck, and biopsy confirmed metastatic serous carcinoma. Ultrasound result showed disseminated multiple enlarged lymph nodes. The patient was diagnosed with platinum-resistant recurrence of ovarian cancer. CA-125 was 1,038 U/mL. After five cycles (from August 2020 to December 2020) of bevacizumab and second-line chemotherapy (paclitaxel + carboplatin), the lymph nodes in the left neck reduced to 0.8 cm, and CA-125 was normal.

**Figure 1:**
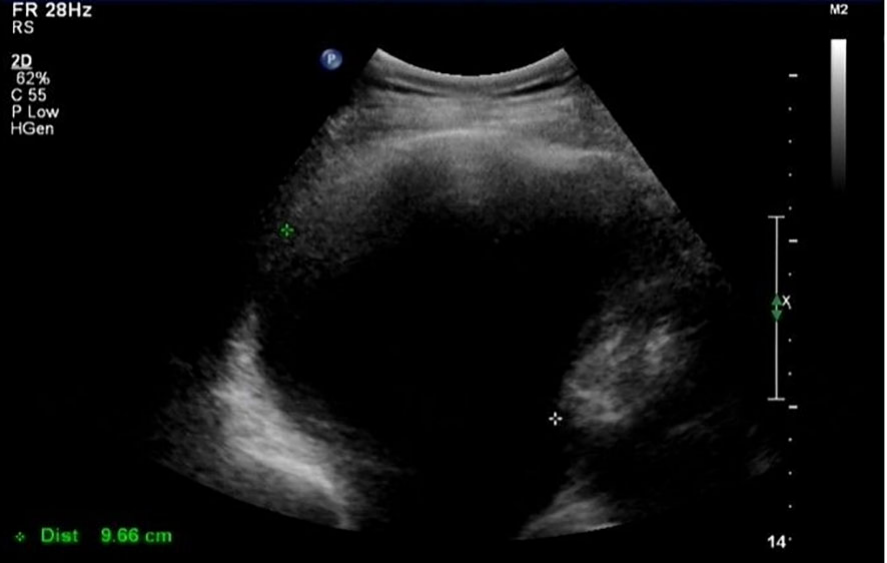
Ultrasound image of pleural effusion.

**Figure 2:**
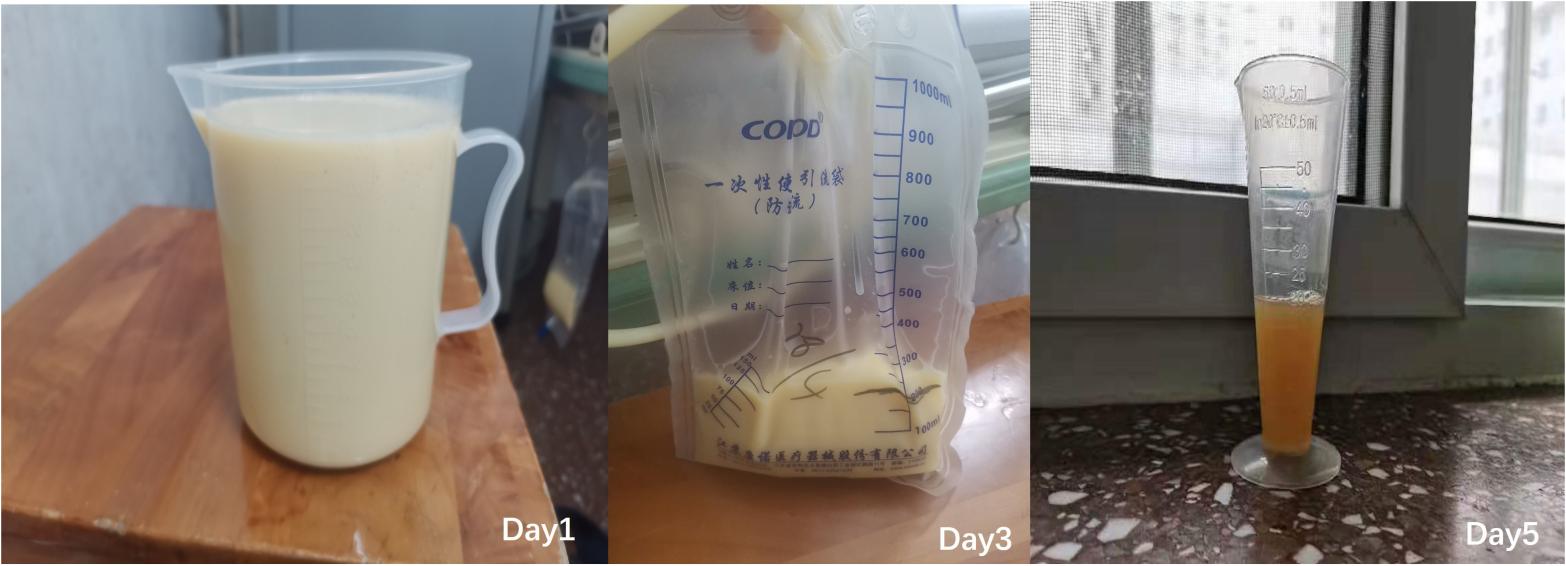
Chylous pleural fluid on day 1, day 3, and day 5.

In March 2021 (3 months after the last chemotherapy), a hard mass that was approximately 5 cm in diameter on the left lower jaw was palpable. CA-125 was 1,863 U/mL. Ultrasound result showed a right pleural effusion (9.8 cm in depth, **Figure 1**). Thoracentesis was performed, and the drainage fluid had a milky white appearance (**Figure 2**). The laboratory analysis of the pleural fluid revealed exudative chylous fluid with triglyceride levels >1.24 mmol/L (110 mg/dL), a cholesterol level <5.18 mmol/L (200 mg/dL), and a positive chyle test. Cytological pathology revealed adenocarcinoma cells. The patient was diagnosed with platinum-resistant recurrence of ovarian cancer combined with right-sided chylothorax. Conservative treatment of chylothorax and chemotherapy for recurrent ovarian cancer were recommended by a multi-disciplinary team, including nutriology, respiratory oncology, thoracic surgery, and intervention departments. Surgery was not feasible because of disseminated multiple metastatic enlarged lymph nodes of ovarian cancer. Chylothorax was managed conservatively, including a strict diet with low-fat medium–long-chain triglycerides and chest tube insertion with interleukin-2 intrapleural perfusion (3 million units). Chylothorax improved significantly with progressively reduced and transparent pleural effusion (**Figure 2**). The drainage fluid was reduced to 50 mL/day on the fifth day, and then the drainage tube was removed. However, long-term intake of medium–long-chain triglycerides affected her quality of life negatively, and the patient was not able to adhere to the diet plan and gradually resumed the regular diet. Therefore, chylothorax recurred, and thoracentesis was performed repeatedly with IL-2 or cisplatinum pleural perfusion at intervals of 2–5 months (a total of six times). Chyle was produced slowly and did not affect the quality of life of the patient. Oral etoposide and bevacizumab were maintained from November 2021 to May 2022 (the patient refused PARP inhibitors due to economic reasons). The patient achieved a partial response (CA-125 91 U/L, lymph node reduction, and pleural fluid maintained at 3.1–5.8 cm).

The patient had repeated tumor recurrence in June 2022, which mainly manifested as increased CA-125 and distant lymph node metastasis (maximum diameter approximately 2–3 cm). Partial remission was achieved after third-line (gemcitabine) and fourth-line (albumin-bound paclitaxel) chemotherapy, targeted therapy, and immunotherapy. The chyme volume in the right thoracic cavity was stable, the patient had no chest congestion or dyspnea, and her nutritional status was fine. The treatment was discontinued due to severe myelosuppression.

**Figure 3:**
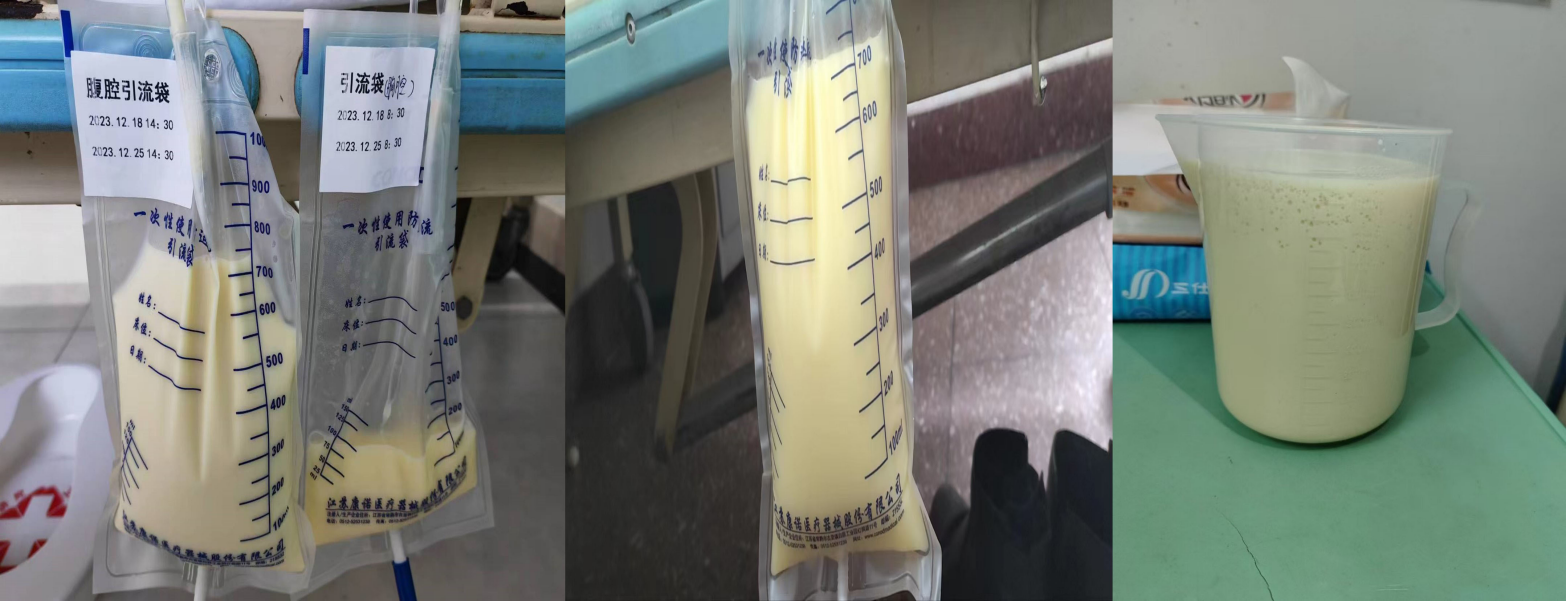
Chylous ascites.

In June 2023, the patient complained of chest tightness and dyspnea. Bilateral pleural effusion was found. The pleural drainage fluid was still chylous. She could not tolerate systemic chemotherapy because of severe myelosuppression. Symptomatic treatments for chylothorax were performed, including thoracic puncture drainage and injection of bevacizumab and IL-2 into the pleural cavity. A large increase in abdominal fluid appeared in December 2023, and the drainage fluid was also chylous (**Figure 3**). Symptoms were partially relieved after perfusion of cisplatin and bevacizumab into the abdomen. The patient died in March 2024.

## 3 Discussion

Chylothorax is a rare condition that results from thoracic duct damage or blockage with chyle leakage from the lymphatic system into the pleural space. Ovarian cancer complicated with chylothorax is rarely reported. Because of its rarity, the characteristics of the disease are not well established in the published works. We reported a case of OCC with a long-term follow-up of 6 years and tried to identify its clinical characteristics.

### 3.1 Etiology of ovarian cancer complicated with chylothorax

The thoracic duct, the largest lymph vessel in the human body, is approximately 36–45 cm long and 2–3 mm wide; it starts from the chylous pool in front of the second lumbar vertebra, enters the thoracic cavity through the aortic hiatus of the diaphragm, courses upward behind the esophagus in the mediastinum, and terminates in the subclavian vein. The primary role of the thoracic duct is to carry 60%–70% of ingested fat from the intestine to the circulatory system. Chyle contains large amounts of cholesterol, triglycerides, chylomicrons, and fat-soluble vitamins. Approximately 1.5–2.5 L of chyle is transported through the lymphatic system every day. Chyle transportation is maximal after a high-fat meal and minimal with starvation (8). Damage to or blockage of the thoracic duct can cause chylothorax.

Tumors and trauma are the most common etiologies of chylothorax (9). 1) Thoracic duct obstruction due to malignant tumor is the most common cause, of which lymphoma accounts for 70%. Rarely can a metastatic lymph node give rise to duct obstruction. In this paper, the patient relapsed mainly with extensive systemic lymphatic metastasis, including mediastinal lymphatic metastasis, which invaded and compressed the thoracic duct and its branches, resulting in chylothorax. Ruchi Gupta reported a similar case of ovarian epithelial malignancy with chylothorax (1). 2) Trauma is the second leading cause, accounting for 23% of chylothorax, which can be further sub-classified as iatrogenic or non-iatrogenic. The incidence of chylothorax caused by esophageal surgery was 4%. However, in ovarian cancer, only two patients were reported to have presented with chylothorax due to a thoracic duct injury after the resection of cardiophrenic lymph nodes through a transdiaphragmatic approach (2, 3). Therefore, the etiology of chylothorax in ovarian cancer includes two aspects: mediastinal lymphatic metastasis invading the thoracic duct and a surgical injury of the thoracic duct during cytoreductive surgery.

### 3.2 Clinical features and diagnosis

A clinical feature of chylothorax is a lack of specificity, depending on the rate of chyle loss. Rapid loss is associated with respiratory distress, such as dyspnea, chest pain, and cough. Patients may experience malnutrition, immunosuppression, and other internal environmental disorders in severe long-term chylothorax.

Chylothorax may be unilateral, either right-sided (50%) or left-sided (33.3%) or bilateral (16.66%), and is dependent on the location of the leak (8). Damage to the duct above the fifth thoracic vertebra results in a left-sided effusion, whereas damage to the duct below this level leads to a right-sided effusion. An injury of the duct between the third and sixth thoracic vertebrae may cause bilateral chylothorax.

The color of chylothorax shows the classical milky white appearance in most cases, but may be serous, yellow, or bloody in appearance in some cases, which may lead to misdiagnosis. Therefore, the exact diagnosis of chylothorax is conducted via thoracentesis and laboratory analysis of the pleural fluid. The diagnostic criteria include the following (10, 11): 1) the presence of chylomicrons in the pleural fluid, 2) a positive chyle test (Sudan III staining method, the presence of red fat globules under the microscope indicates a positive result), and 3) pleural fluid triglyceride levels >1.24 mmol/L (110 mg/dL) with a cholesterol level <5.18 mmol/L (200 mg/dL). Pseudochylothorax presenting a milky white appearance should be differentiated (8, 11, 12). A triglyceride level <0.56 mmol/L (50 mg/dL) with a cholesterol level >5.18 mmol/L (200 mg/dL) can be found in pseudochylothorax. Cholesterol crystals are also often seen in pseudochylothorax but are not present in chylothorax. Chylothorax can be differentiated from pseudochylothorax by adding 1–2 mL of ethyl ether. The milky appearance disappears in pseudochylothorax. Maldonado et al. (13) analyzed protein in pleural fluid and found that pleural effusions were exudative in 86% of cases and transudative in 14%. Exudative chylothoraces were found in patients with malignancy, while transudative chylothoraces were found in patients with cirrhosis, surgery, and pancreatic cancer.

CT of the thorax and abdomen should be performed to identify the amount of chylothorax and its relation to malignant tumors. In this case, multiple mediastinal enlarged lymph nodes could be clearly seen on CT, leading to chylous pleural fluid formation. Lymphangiography may be used to demonstrate the site of leakage or blockage. Matsumoto et al. (14) performed lymphangiography on nine patients who were unlikely to respond to conservative measures. They found that lymphangiography not only identified the site of the leak but also led to the leak resolving in all cases. They recommend early lymphangiography only in cases unlikely to be treated by conservative methods.

### 3.3 Treatment and prognosis

Compared to pleural fluid in ovarian cancer, it is difficult to treat chylothorax. The treatment of OCC includes three aspects: anti-tumor treatment of ovarian cancer and its metastasis, conservative management, and surgical management of chylothorax.

Anti-tumor treatment plays an important role in treating OCC. The patient in this case relapsed with extensive systemic lymph node metastasis of ovarian cancer, and chylothorax was formed due to an enlarged metastatic mediastinal lymph node and invasion of the thoracic duct. Therefore, the patient had to accept chemotherapy. Multi-line chemotherapy plus bevacizumab brought benefits for controlling ovarian cancer overall, reducing the metastatic lymph nodes, as well as leading to an improvement in chylothorax.

Conservative treatment of chylothorax involves the following: 1) medium- and long-chain triglycerides are directly absorbed into the portal system, bypassing the intestinal lymph system and thoracic duct. The diet of low-fat medium–long-chain triglycerides can reduce the flow of chyle in the thoracic duct and resolve approximately 50% of chylothorax (11). If a chyle leak does not reduce yet, then total parenteral feeding should be considered. 2) Somatostatin and octreotide, which can reduce intestinal chyle production, have been proven to be useful in the conservative treatment of chylothorax (15). 3) Draining pleural effusion using thoracentesis should be conducted to resolve respiratory distress and ensure complete lung expansion. At the same time, pleurodesis agents including talc, tetracycline, bleomycin, povidone, and elemene have been successfully used to produce chemical pleurodesis in the majority of patients (16, 17).

In this paper, we adopted a combined treatment plan consisting of anti-tumor therapy and conservative treatment of chylothorax. In more than 2 years, the patient had no or slight chest tightness or dyspnea, right-sided chylothorax was stable, and her nutritional status was fine. Nevertheless, chyle did not disappear completely, which is consistent with literature reports (16). However, Cowan and Helene (2, 3) reported two cases of chylothorax caused by damage to the thoracic duct during ovarian cancer surgery, which were treated after conservative management with fat-free enteral nutrition for 6 weeks. Based on this, we believed that, unlike chylothorax caused by trauma, conservative treatment is effective but has a low success rate in treating chylothorax caused by tumor blockage of the thoracic duct. Due to severe myelosuppression, the antineoplastic treatment was stopped, and the disease progressed with bilateral chylothorax and chylous ascites, which eventually led to the patient’s death.

Surgery is recommended if the drainage fluid exceeds 1.5 L/day or more than 1.0 L/day for five consecutive days or continues to drain for more than 2 weeks after conservative treatment (15). Surgical options include thoracic duct percutaneous embolization, surgical ligation of the thoracic duct, and pleuroperitoneal shunt (18). Thoracic duct ligation is advocated when chylothoraces are caused by an operation, especially esophageal surgery. The key point of thoracic duct ligation is identifying the origin of a chyle leak. However, extensive dissection to find the origin is discouraged in order to reduce further trauma. In the two cases of OCC, the authors failed to identify the origin of the chyle leak and were not able to perform thoracic duct ligation during thoracotomy (2, 3). Therefore, we do not recommend thoracic duct ligation first when chylothorax is caused by metastatic enlarged lymph nodes in patients with OCC.

The mortality rate for cases of chylothorax is approximately 10%; chylothoraces caused by thoracic duct obstruction due to mediastinal malignancies have the worst prognosis (19). Because of the rarity of OCC, the prognosis is unclear yet. Gupta R reported a case of ovarian epithelial malignancy with chylothorax in which the patient ultimately succumbed to the illness (1). The patient in this case died after 6 years of comprehensive treatment. Due to the rarity of ovarian cancer with chylothorax, the prognosis is unknown.

Taken together, OCC is a rare condition with a relatively poor prognosis. Pleural fluid analysis can identify this condition when clinical suspicion exists. Anti-tumor treatment of ovarian cancer and conservative management of chylothorax are first recommended, which have been proven to be effective in treating OCC. 

## Data Availability

The original contributions presented in the study are included in the article/supplementary material. Further inquiries can be directed to the corresponding author.
